# Resolution of American Medical Association

**Published:** 1887-09

**Authors:** 


ARTICLE V.

### RESOLUTION OF AMERICAN MEDICAL ASSOCIATION.

The American Medical Association, which met in Chicago recently, passed the following resolutions:

RESOLVED, That the regular graduates of such dental and oral schools and colleges as require of their students a standard of preliminary or general education, and a term of professional study equal to the best class of the medical colleges of this country, and embrace in their curriculum all the fundamental branches of medicine, differing chiefly by substituting practical and clinical instruction in dental and oral medicine and surgery, in place of practical and clinical instruction in general medicine and surgery, be recognized as members of the regular profession of medicine, and eligible to membership in this association on the same conditions and subject to the same regulations as other members.

Dr. Davis in introducing the resolution said he wished to explain its object. "There are two objects to be had in view; first, to relieve a degree of embarrassment that exists between the regular profession as we consider it, and the profession of dentistry. The department of dental and oral surgery is a part of the profession of medicine as much as the department of ophthalmology or otology or any other ology. Our teeth and mouths are a part of our system as much as any other part, and are used more than any other part. The embarrassment is this: That in history the development of dentistry originated mostly in mechanical operations. Steadily it has advanced and in years gone by—quite a number of years ago—our lamented S. D. Gross made a proposition that an oral and dental section be provided as a section in this association. It was seconded by Dr. Sayre and myself, and it was organized. The International Medical Congress of 1881 provided a section for dental and oral surgery. The congress to be held in Washington has done the same thing, and it will be one of the most thorough and best organized sections in the congress. There is an embarrassment in this respect. It is to know just who and by what line of demarkation those engaged in that department shall be recognized as members of the regular profession. Now it is proposed to make a line and draw it where this resolution says that all those who are qualified by general education and a term of study equal to the best medical colleges, a curriculum embracing the entire fundamental principles of medicine with the provision that instead of special instruction in clinical surgery, instruction may be had in dental and oral surgery, such shall be recognized as members of the profession of medicine. It will take away a sort of embarrassment. There is a more far-reaching and more valuable underlying object in this resolution, ana that is that to be recognized as a member of the profession, if this resolution is adopted by this body, they must have the education received in schools that require these requirements, it makes a strong lever to lift up the course of study in the dental schools. Such are my reasons for bringing up the resolution. I will say nothing more on the subject."

The motion was made that the resolution be adopted by the association, and it was carried unanimously.—*Ez.*

